# microRNAs and next generation sequencing for the prognosis of the metastatic melanoma

**DOI:** 10.1186/1479-5876-13-S1-P4

**Published:** 2015-01-15

**Authors:** Rosamaria Pinto, Simona De Summa, Sabino Strippoli, Gabriella Guida, Ondina Popescu, Amalia Azzariti, Michele Guida, Stefania Tommasi

**Affiliations:** 1 IRCCS Istituto Tumori “Giovanni Paolo II”- Molecular Genetics Laboratory- Bari, Italy; 2 IRCCS Istituto Tumori “Giovanni Paolo II”- Oncology Unit- Bari, Italy; 3 University of Bari- Department of Medical Biochemistry – BARI, Italy; 4 IRCCS Istituto Tumori “Giovanni Paolo II”- Anatomopathology Unit- Bari, Italy; 5 IRCCS Istituto Tumori “Giovanni Paolo II”- Clinical and Preclinical Pharmacology Laboratory- Bari, Italy

## Background

Melanoma is the most serious form of skin cancer because of its increasing incidence. Recent studies demonstrated the involvement of specific microRNAs in the melanoma initiation, progression, diagnosis and prognosis. Currently the treatment for metastatic melanoma with the mutation of BRAF V600E expects the Vemurafenib that blocks mutated BRAF protein leading to cell-cycle arrest. Unfortunately, the reactivation of MAPK signalling or activation of an alternative signalling pathway, as PI3K/AKT/mTOR, deriving from different mechanisms of acquired tumor drug resistance (as secondary mutations in NRAS or MEK1) causes disease progression within 6–8 months after the therapy beginning. The aim of this work was to evaluate a possible MAPK reactivation due to microRNAs involvement or to unclassified gene variants not yet associated to metastatic melanoma therapy response.

## Materials and methods

A set of 43 patients, treatment naïve and with confirmed histological stage IV of metastatic melanoma was enrolled through the Oncology Unit of the IRCCS “Giovanni Paolo II” in Bari, Italy. Thirty melanoma cases were BRAF mutated at the codon 600, while 13 were wild type. We analyzed 15 microRNAs and the correspondent target genes by TaqMan probes. Moreover we developed an Ampliseq Custom panel for ION Torrent PGM Sequencer to analyze the coding region of several target genes with a coverage of 93.85%. The correlation between microRNA expression signature and the detected mutations with time to progression of patients treated with Vemurafenib has been analyzed.

## Results

High expression of miR-192 and miR-193b* and low expression of miR-132 resulted associated with short time to progression, by the Kaplan-Meier survival curves, indicating a poor prognosis. MiR-193b*was also included in the results of the multivariate Cox analysis that revealed a significant signature of 11 microRNAs (miR-34a, miR-146b, miR-182, miR-155, miR-101, miR-222, miR-21, miR-338-3p, miR-193b*, miR-193a and miR-191) whose high expression was associated to worse prognosis. The univariate Cox model confirms that potential role for miR-193b* as poor outcome marker. Moreover we detected mutations associated with the response to different therapeutic approach and, more interestingly, we identified gene alterations not yet associated to this pathology.

## Conclusions

Our study highlighted the prognostic role of microRNAs in metastatic melanoma. Furthermore the use of Next Generation Sequencing to detect known or novel mutations could be useful in clinical practice.

